# Mathews’ List of Drugs, Chemicals, Etc., and Price Current

**Published:** 1859-01

**Authors:** 


*Mathews' List of Drugs, Chemicals, etc., and Prices Current.*—With this issue we publish a catalogue of the drugs and chemicals which are to be found in the extensive establishment of Mr. A. I. Mathews, and the current prices. This will appear with each number, with such modification as may become necessary. By this means, our readers will be able to accertain what medicines may be procured in Buffalo, and at what prices; and even should they not desire to purchase them here, they will be always posted in regard to the market price. This, we conceive, will be a very useful feature of the Journal, and it is intended to make it a permanency.

